# Enteroviral infections are not associated with type 2 diabetes

**DOI:** 10.3389/fendo.2023.1236574

**Published:** 2023-10-30

**Authors:** Huan Liu, Shirin Geravandi, Ausilia Maria Grasso, Saheri Sikdar, Alberto Pugliese, Kathrin Maedler

**Affiliations:** ^1^ Centre for Biomolecular Interactions Bremen, University of Bremen, Bremen, Germany; ^2^ The JDRF nPOD-Virus Group; ^3^ Diabetes Research Institute, Department of Medicine, Division of Endocrinology and Metabolism, Miami, FL, United States; ^4^ Department of Microbiology and Immunology, Leonard Miller School of Medicine, University of Miami, Miami, FL, United States; ^5^ Department of Diabetes Immunology & The Wanek Family Project for Type 1 Diabetes, Arthur Riggs Diabetes & Metabolism Research Institute, City of Hope, Duarte, CA, United States

**Keywords:** type 2 diabetes, enterovirus, coxsackievirus, islets, pancreas

## Abstract

**Introduction:**

For more than a century, enteroviral infections have been associated with autoimmunity and type 1 diabetes (T1D). Uncontrolled viral response pathways repeatedly presented during childhood highly correlate with autoimmunity and T1D. Virus responses evoke chemokines and cytokines, the “cytokine storm” circulating through the body and attack cells especially vulnerable to inflammatory destruction. Intra-islet inflammation is a major trigger of β-cell failure in both T1D and T2D. The genetic contribution of islet inflammation pathways is apparent in T1D, with several mutations in the interferon system. In contrast, in T2D, gene mutations are related to glucose homeostasis in β cells and insulin-target tissue and rarely within viral response pathways. Therefore, the current study evaluated whether enteroviral RNA can be found in the pancreas from organ donors with T2D and its association with disease progression.

**Methods:**

Pancreases from well-characterized 29 organ donors with T2D and 15 age- and BMI-matched controls were obtained from the network for pancreatic organ donors with diabetes and were analyzed in duplicates. Single-molecule fluorescence *in-situ* hybridization analyses were performed using three probe sets to detect positive-strand enteroviral RNA; pancreas sections were co-stained by classical immunostaining for insulin and CD45.

**Results:**

There was no difference in the presence or localization of enteroviral RNA in control nondiabetic and T2D pancreases; viral infiltration showed large heterogeneity in both groups ranging from 0 to 94 virus^+^ cells scattered throughout the pancreas, most of them in the exocrine pancreas. Very rarely, a single virus^+^ cell was found within islets or co-stained with CD45^+^ immune cells. Only one single T2D donor presented an exceptionally high number of viruses, similarly as seen previously in T1D, which correlated with a highly reduced number of β cells.

**Discussion:**

No association of enteroviral infection in the pancreas and T2D diabetes could be found. Despite great similarities in inflammatory markers in islets in T1D and T2D, long-term enteroviral infiltration is a distinct pathological feature of T1D-associated autoimmunity and in T1D pancreases.

## Graphical Abstract

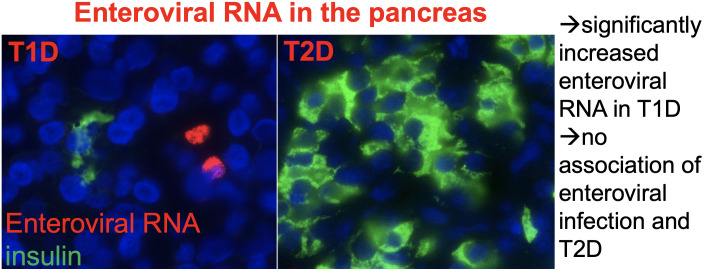

## Highlights

- Enteroviral RNA^+^ cells were found scattered throughout the whole pancreas from organ donors.
- They were very rarely seen within islets.
- Enteroviral RNA^+^ cells were similar in controls and T2D donors.
- CD45^+^ lymphocytes were increased and β-cell area decreased in the pancreases in T2D.
- In contrast to T1D, enteroviral infiltration is not a pathological feature of T2D pancreases.

## Introduction

Ever since Cerasi and Luft have recognized that type 2 diabetes (T2D) is caused by the relative inability of β cells to secrete sufficient amounts of insulin to compensate for insulin resistance, and not by insulin resistance itself (1), underlying mechanisms of this β-cell defect have been searched for and found quite complex, with β-cell inflammation as a major factor in both T1D (2) and T2D (3, 4). Low-grade inflammation is detectable in serum (5) and in single cells within the islets in the pancreas (3, 6) and correlates with accelerated β-cell loss in T2D (7).

Although there is little sign of an acute viral infection in the pancreas, the association of enteroviruses and autoimmune disease, especially T1D (8), has been identified through highly sensitive methods and analyses of carefully collected patients’ material in the context of well-powered studies (9–11). The increased presence of enteroviral RNA in the pancreas from organ donors with T1D has just been confirmed in a large meta-analysis (12). Especially, long-duration or multiple enterovirus B (EVB) infections correlate with islet autoimmunity and T1D progression (13, 14). Isolated enteroviruses from human pancreas obtained via biopsy near the T1D onset could be transmitted to cells in culture and produced an immune response (15). Enteroviruses can persist in the pancreas and chronic and/or repeated infection results in the production of inflammatory mediators and triggers an inflammatory response against islet cells (16). The virus is suggested to carry a deletion at the 5′ terminus that renders it persistent and non-cytopathic (17). Using vaccines against coxsackieviruses group B, preclinical studies have successfully prevented infection and CVB-induced diabetes, and clinical studies are in progress (18).

Stark stimulation of viral response pathways seems to foster autoimmune as well as metabolic disease, as seen during the SARS-CoV-2 pandemic, where T1D onset after COVID-19 was identified in several (19, 20) but not all studies (21). More apparent is the highly increased severity of COVID-19 in patients with T2D and obesity in one hand and progression of T2D after COVID-19 on the other (22, 23), with circulating chemokines and cytokines orchestrating a “cytokine storm” that impacts multiple organs in the body. Cells especially vulnerable to inflammatory attack are destroyed and viral response pathways uncontrolled (24) (9, 25),.

Inflammation is a major trigger of β-cell failure, loss of function and apoptosis, both in autoimmune T1D and T2D (4). Islet inflammation as a primary modulator of the progression of T2D had initially been challenged (e.g., reviewed here (26, 27), but has been confirmed by numerous studies from different laboratories and now achieved its acceptance in textbooks (e.g. (28)). Numerous environmental factors such as not only viral infection but also chronic stress, overnutrition which leads to “gluco- and lipotoxicity,” islet amyloid and islet amyloid polypeptide (IAPP) toxic oligomer disposition in islets and bacterial LPS alone or in concert lead to islet inflammation (29–34). Also, other mechanisms of β-cell failure in T1D and T2D, such as dedifferentiation and loss of identity, have been suggested to result from inflammatory insults (35, 36).

Significantly higher protein and mRNA levels of cytokines and chemokines such as IL-1β, IL-6, IL-8, IP-10, IL-17, and MCP1 (3, 6, 27, 32, 37, 38) together with macrophage infiltration have been identified in islets; alterations were observed in *in-vitro* and *in-vivo* models of T2D, in isolated islets and in autopsy-pancreases from donors with T2D. These changes of immune components, specific cytokines, and chemokines, and the occurrence of apoptosis, confirm that an inflammatory response is involved in the pathogenesis of T2D (39, 40).

The genetic contribution to islet inflammation pathways is apparent in T1D, with polymorphisms in the interferon system (41, 42) and interferon and viral infection signatures identified in islets of organ donors with T1D (43, 44). Even in T2D, where gene mutations are related to glucose homeostasis in β cells and insulin target tissues (45), genetic variants affecting viral response pathways have been identified; for example, a TYK2 promoter variant associated with a deteriorated cytokine response has been identified as risk factor for T1D as well as T2D (46) and correlates with increased T2D severity (47).

As coxsackievirus infection is associated with β-cell dysfunction and apoptosis (17, 48), a connection with T2D has been hypothesized. The European Prospective Investigation of Cancer-Norfolk study investigated the association between infection, coxsackievirus B serotype 1–5 seropositivity, and T2D, but no correlation between coxsackievirus B neutralizing antibodies and T2D has been found (49). The presence of the enterovirus-specific viral capsid VP1 within islet cells has been found more often in pancreases of patients with T1D than in those with T2D, and only rarely in nondiabetic controls (48, 50). Despite these previous studies, it remained unclear whether there is indeed more enteroviral disposition in the pancreas associated with T2D. We therefore applied a deep and thorough analysis of enteroviral RNA by high-sensitivity single-molecule fluorescence *in-situ* hybridization (FISH), which had been previously demonstrated increased viral RNA in the pancreas of patients with T1D and with islet associated autoimmunity (51), to the network for pancreatic organ donors with diabetes (nPOD) collection of well-characterized pancreases from organ donors with T2D and their age- and BMI-matched controls (52).

## Material and methods

### Pancreas autopsy material

This study used formalin-fixed paraffin-embedded (FFPE) pancreatic tissue sections obtained from well-characterized organ donors from the nPOD. Donors included 29 with T2D (average disease duration 9 years, range 0.25–26 years, and 15 control donors (without diabetes; **Supplementary Table S1**). Mean donor age for both groups is 51 years and mean BMI is 28.5 (controls) and 32.9 (T2D). Results were compared to a previous analysis of 15 organ donors with T1D from nPOD and their nondiabetic controls (14; mean donor age 22 years, BMI 24 and 25, respectively) (51).

### Virus detection in FFPE tissue samples

Custom Stellaris^®^ FISH Probes against enteroviral RNA labeled with Quasar 570 were used to detect viral RNA as described previously (Biosearch Technologies, Inc., Petaluma, CA, USA) (51, 53). The three probe sets recognize various enteroviral strains for positive-strand enteroviral RNA, CVB_1 was designed on the CVB3 consensus-based sequence (M33854.1), 106 genome sequences of the enterovirus group B family enteroviruses were aligned, and sequences were then divided into three subgroups based on sequence similarities (CVB_1, CVB_2, and CVB_3) (54). The following stepwise previously established highly sensitive protocol (53) was performed for enterovirus mRNA detection by smFISH in pancreatic tissue sections:

Deparaffinization of FFPE tissue sections. Removal of paraffin by a series of Xylene washes (20 min at 70°C; 10 min at 70°C; 10 min at room temperature), followed by rehydration by ethanol (EtOH; 100%, 100%, and 95%) for 10 min each and for 1h in 70% EtOH at room temperature and rehydrating with RNase free water 2 times for 2 min, all under constant steering.

Prehybridization. Incubation with 0.2M HCl for 20 min at room temperature, transfer to a 50-ml tube with prewarmed 2xSSC and incubation at 70°C for 15 min, phosphate-buffered saline (PBS) 2 times for 2 min at room temperature, incubation with 37°C pepsin (Sigma-Aldrich) for 10 min, washing 2 times with PBS for 1 min and with 0.5% Sudan Black (Sigma-Aldrich) in 70% EtOH for 20 min at room temperature to quench remaining autofluorescence, followed by serial washings with PBS and washing buffer (1xSSC,10% formamide).

Hybridization. Three probes were diluted 1:100 in hybridization buffer (10% w/v dextran sulfate, 10% formamide, 2xSSC) and applied to the sections, glass-covered and incubated at 37°C for 12h–14h in a humidified chamber.

Post-hybridization wash. Cover slips were removed by hybridization buffer, sections washed in 37°C prewarmed solutions: 2 times 2xSSC + 10% formamide for 20 min, 2 times with 2xSSC for 15 min, followed by 2 times wash with 1xSSC for 15 min, then with 0.1xSSC for 15 min and, last, with 0.1xSSC for 5 min.

Thereafter, classical immunostaining was performed for insulin (Dako#A0546), the general lymphocyte marker CD45 (Dako#M0701) and VECTASHIELD^®^ antifade mounting medium (Vector laboratories) including 4′,6-diamidin-2-phenylindol (DAPI). A 60× oil-immersion objective was used to acquire images images by a Nikon Ti MEA53200 (NIKON GmbH, Düsseldorf, Germany) microscope.

### Quantification of cells and tissues

Morphometrical analyses of enteroviral mRNA, insulin, and CD45 were performed with a NikonTiMEA53200 (NIKON GmbH, Düsseldorf, Germany) microscope and NIS-Elements BR software. The number of virus-infected cells and number of islets and immune cells were counted manually throughout the whole sections. Viral RNA appeared as small dots within cells, which were separately counted for each cell by moving the z-focus of the microscope through each virus^+^ cell. Cells were defined as “single infected” with 1–10 puncta or “fully infected” with ≥10 puncta. A representative picture of infected cells was taken in a way that most “puncta” could be seen. Mean β-cell area per pancreas was calculated as the ratio of insulin-positive to whole pancreatic tissue area. The exocrine area was calculated as whole pancreas area subtracted by the insulin-positive area. “Islet periphery” was defined as signal localization within three cell layers next to insulin containing islets and “close proximity” as signal localization within three cell layers next to the respective islet or immune cells.

### Statistical analyses

All biological replica referred to “n” for each individual human pancreas, which are means of two technical replicas from independent staining analyses and presented as means ± SEM. Mean differences were determined by the Mann–Whitney non-parametric two-tailed test, in which the whole T2D group was compared to the control group without diabetes. In a subgroup analysis (**Supplementary Figure S3**), either the Aab^–^T2D group or the Aab^+^-T2D group was compared to the control group without diabetes. A *p*-value <0.05 was considered statistically significant. Investigators were blinded to the cases.

### Study approval

Ethical approval for the use of human pancreatic tissue had been granted by the Ethics Committee of the University of Bremen. The study complied with all relevant ethical regulations for work with human tissue for research purposes. Organ donors or next of kin provided written informed consent for organ donation for research (52).

## Results

**Figure 1:**
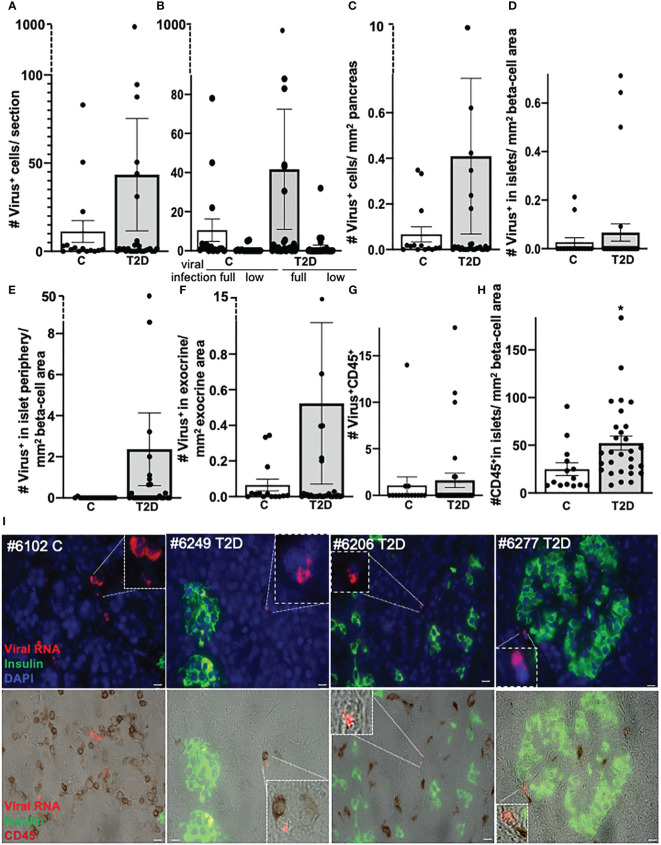
No correlation of pancreatic enteroviral RNA in organ donors with T2D and controls without diabetes. Detection and quantification of viral RNA in FFPE pancreases from control donors without diabetes (*n* = 15) and donors with T2D (*n* = 29). Data are presented as **(A)** mean number of all enteroviral RNA^+^ cells throughout the whole pancreas section, **(B)** the mean number of low grade (low; one to nine single puncta/cell) or full-grade (full; ≥10 single puncta/cell) infected cells. In the latter, viral RNA highly accumulated in the cell and therefore could no longer be counted as single puncta. **(C)** All enteroviral mRNA^+^ cells were normalized to the whole pancreas area of the respective section. **(D–F)** Enteroviral mRNA^+^ cells within islets **(D)** and within the periphery of three cells next to insulin containing islets **(E)** were normalized to islet area (insulin^+^ stained area in mm^2^), and **(F)** viral mRNA^+^ cells in the exocrine area were normalized to the mm^2^ exocrine area of the respective section. **(G)** Quantification of enteroviral RNA^+^/CD45 co-positive cells throughout the whole pancreas section and **(H)** of CD45^+^cells within insulin containing islets normalized to mm^2^ islet area. Each individual point of the scatter graphs represents the mean of two technical replica from each donor pancreas, boxes are means ± SEM from all donors. *P < 0.005 by Mann–Whitney non-parametric two-tailed test. **(I)** Representative microscopical pictures of enteroviral RNA in the pancreas. Quadruple immunostainings of enteroviral RNA (red), insulin (green), DAPI (blue, all upper), and CD45 (brown, lower) in FFPE pancreases from a control donor without diabetes (1) and three donors with T2D (2–4) and their localization within the exocrine pancreas (1–3) or within the periphery of three cells next to insulin containing islets (4) and their proximity to CD45^+^ lymphocytes. Scale bars depict 10 µm. Magnifications show enteroviral RNA^+^ cells.

**Figure 2:**
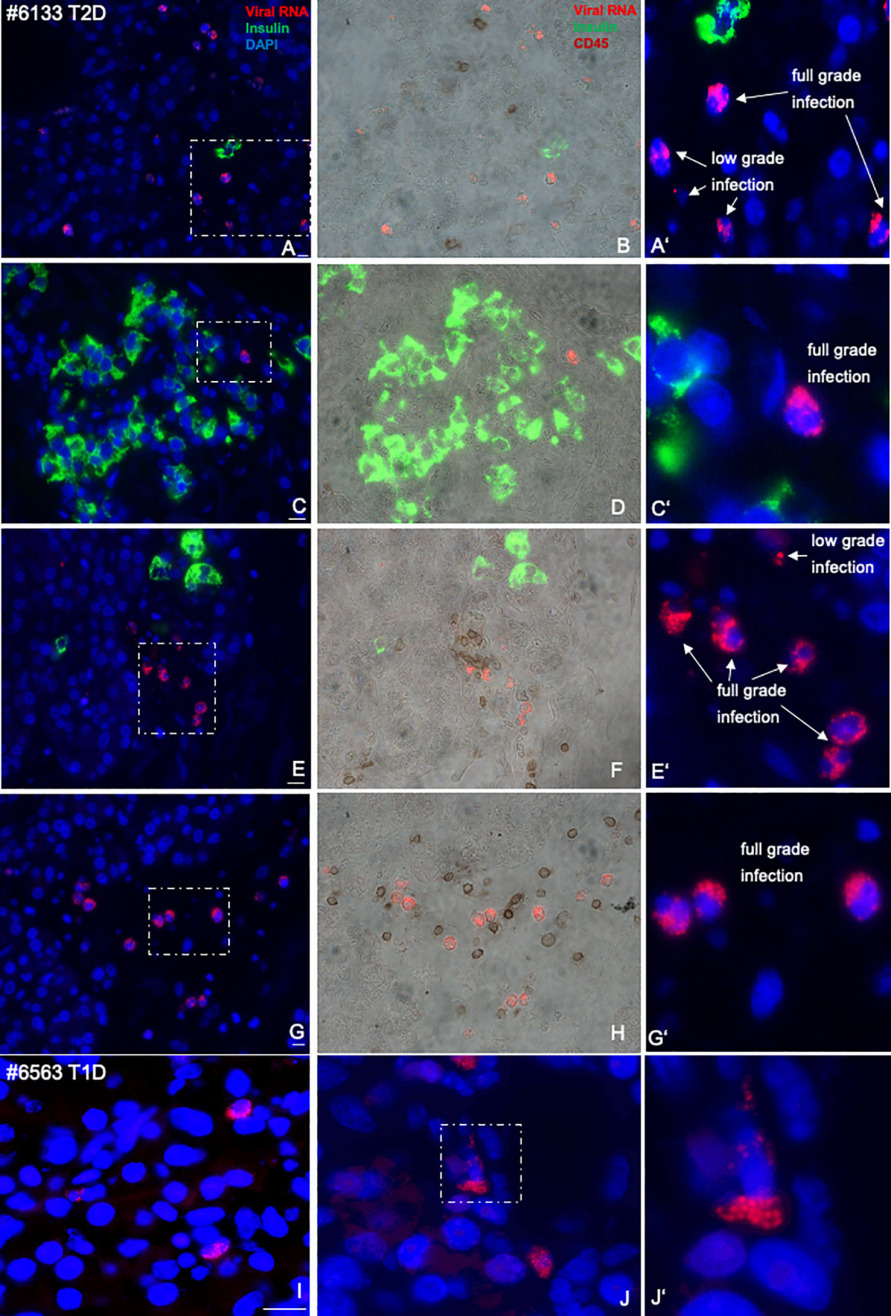
Representative microscopical pictures of pancreas sections from an nPOD donor with T2D with specifically high-enteroviral RNA and comparison with T1D. Quadruple immunostainings of enteroviral RNA (red), insulin (green), DAPI (blue, left **(A, C, E, G)** and CD45 (brown, middle **(B, D, F, H)** in an FFPE pancreas from a donor with T2D scattered throughout the pancreas in proximity to single β cells **(A, B)**, islets **(C, D)**, or scattered within the pancreas **(E–H)**, in proximity to CD45^+^ lymphocytes or co-stained with CD45 **(B, F, H)**. Magnifications **(A′, C′, E′, F′)** show enteroviral RNA^+^ cells with low- and full-grade infections, where viral RNA highly accumulated in the cell. Representative pictures of the exocrine region from a donor with T1D was included for comparison **(I, J, I’)** in larger magnification. Representative pictures of infected cells were taken in a way that most “puncta” could been seen. Scale bars depict 10 µm.

Viral infiltration showed large heterogeneity and found scattered throughout the pancreases with no significant difference between controls and T2D (**Figures 1A–C**, **I**; **Supplementary Table S2**). Only one single T2D donor presented an exceptionally high number of virus-expressing cells within the exocrine pancreas, reminiscent of viral infiltration in T1D (**Figure 2**) (51). The proportion of donors with cells harboring virus RNA within the pancreas were 73% (11 of 15) among controls, and 66% (19 of 29) among T2D donors. A direct comparison of the cohort with our previous analysis with organ donors with T1D (51) showed the difference in viral RNA in T2D and T1D (**Supplementary Figure S1**). Despite some heterogeneity in the numbers of infected cells, donors with T1D were all positive for viral RNA^+^ cells in their pancreases (100%), and their quantification showed sevenfold more viral RNA^+^ cells in T1D than in controls of this cohort.

Enteroviral RNA^+^ cells were only very rarely seen in islets (one single cell in two control donors and in 4 T2D donors, respectively; **Figure 1D**). The observation that enteroviral^+^ cells scattered throughout the whole pancreas was verified by normalizing virus^+^ cells within (**Figure 1D**) or in the periphery of islets (**Figure 1E**) to β-cell area and virus^+^ cells in the exocrine pancreas area to exocrine area (**Figure 1F**), all of which were similar in controls and T2D donors (**Figures 1D–F**).

Thereby, the islet periphery was defined as insulin^-^ cells within three layers next to insulin^+^ cells of the islets, in analogy to our previous study in T1D pancreases (51). In these three cell layers, we found many enteroviral^+^ cells in T1D (51), but only few in T2D (**Figure 1E**).

Lymphocytes expressing viral RNA (virus^+^/CD45^+^ co-positive) were rare and had similar frequency in controls and T2D (**Figure 1G**). Interestingly, many CD45^+^ lymphocytes were found in close proximity to a virus^+^ cell in the exocrine area, suggesting an active immune process where virus^+^ cells were recognized by immune cells (**Figure 1I**). However, as this study is limited to the use of fixed tissue, we were unable to verify such active process.

**Figure 3:**
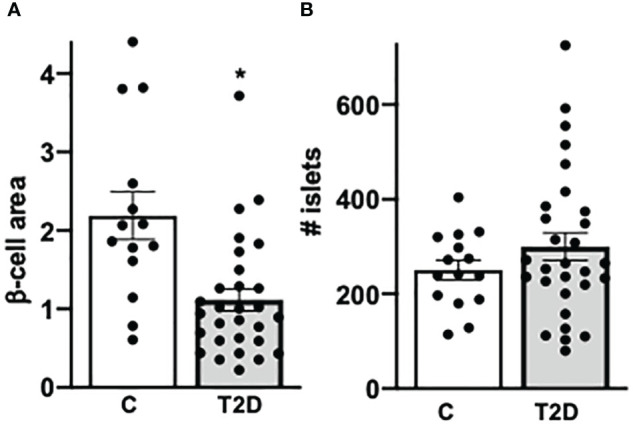
Confirmed decreased β-cell area in T2D. **(A)** For β-cell area analysis, the percentage of β cells were calculated by the ratio of mm^2^ insulin area and mm^2^ of the whole pancreas area from each section (previously also called β-cell volume). **(B)** The absolute number of islets was counted in each section. Each individual point of the scatter graphs represents the mean of two technical replica from each donor pancreas, boxes are means ± SEM from all donors. *P < 0.05 by Mann–Whitney non-parametric two-tailed test.

With the normalization of viruses to their cellular location and the quantification of CD45^+^ lymphocytes and insulin^+^ β cells in the pancreas we confirmed and verified the increase in lymphocytes and the reduction in β-cell area in T2D in this well characterized nPOD cohort (**Figure 1H**), in line with previous elegant studies (6, 7, 55–57). While there was heterogeneity among islets as well as among donor pancreases, β-cell area/exocrine area (previously also called β-cell volume) was reduced by 49% in the pancreases of donors with T2D, compared to controls (**Figure 3**), in line with results obtained from the Mayo clinic’s cohort (7). The number of CD45^+^immune cells localized in islets was twofold increase in T2D donors compared to controls without diabetes, analogously to previous observations in isolated islets (55) and in pancreas sections (57). With an average of 0.3 ± 0.03 CD45^+^ cells per islet, islet lymphocytic infiltration in all donor pancreases of this study (including the donor with the exceptional high number of viruses; **Figure 2**) was much lower than the defined threshold of 15 CD45^+^ cells/islet for T1D (58, 59), which confirms classification to T2D of cases analyzed in this study, despite the higher number of inner-islet-CD45^+^ cells in T2D, compared to nondiabetic controls.

We found no correlation of enteroviruses with the mean number of islets, nor with β-cell area, nor with age of the donor (**Supplementary Figure S2**). Only the single donor with an exceptionally high virus counts of 925 virus-expressing cells throughout the pancreas had the lowest number of islets (mean of 80) in the observed pancreas sections (donor ID #6133; **Figure 2**; **Supplementary Figures S1**, **S2**), together with a low β-cell area (**Supplementary Figure S2B**). Most donors with a relatively high virus count of >20 enterovirus expressing cells throughout the pancreas section were in an age group >45 years (**Supplementary Figure S2C**; dashed box). Only the youngest donor of the whole cohort had a high virus count of 94.5, was already diagnosed with T2D at the age of 15, is severely obese (BMI of 37), and presented uncontrolled hyperglycaemia with an HbA1c of 10.7%. We compared these results with those of younger control donors from our previous analysis (51) (mean age of 21.5 years), and they also showed the very low number of <20 enterovirus^+^ cells/section (**Supplementary Figure S1C**).

Of note, within the T2D group, we have also tested donors that had developed single T1D-associated antibodies against insulin (mIAA; *n* = 10, mean age = 47; **Supplementary Table S1**), most likely in response to subcutaneous insulin injection, as all T2D-IAA^+^ donors had received insulin therapy before or during hospitalization. Two donors were positive for glutamic acid decarboxylase (GADA; *n* = 2; mean age = 53), which is an early T1D-autoantibody and marker of T1D progression (60), but all these donors fulfilled the classification of T2D, based on pancreas morphology and c-peptide levels (59, 61). As we had previously seen a significant correlation of T1D-associated autoantibody positivity and enteroviral RNA in the pancreas in young donors (mean age of 20 years; maximum 26 years), we performed a sub-analysis of this category (**Supplementary Figure S3**) but could not find any significant difference in pancreatic viral RNA and their localization within the pancreas, when compared to controls (**Supplementary Figures S3A–D**). In contrast to our previous study, single AAb^+^ donors, which have developed T2D, did not show any differences in the number of enteroviral RNA in the pancreas, while young single and multiple AAb^+^ donors (mean age of 20 years) without diabetes had a significantly increased enteroviral RNA in the pancreas, compared to nondiabetic controls. Here, only older donors (mean 48 years) with a single GADA- or IAA-AAb were analyzed, who had not progressed to classical T1D or **l**ate onset autoimmune diabetes in adults, their pancreases had many large islets and very few lymphocytes in islets (mean of 0.1 CD45^+^cell/islet) and normal C-peptide levels (mean = 4.3 nmol/L). The increase in lymphocytes (**Supplementary Figure S3E**) and the reduction in β-cell area (**Supplementary Figure S3F**) were again confirmed in T2D, also when each of the two T2D groups with or without T1D-associated Aabs were independently compared to the control pancreases without diabetes.

## Discussion

Altogether, in T2D, enteroviral RNA could be detected within the pancreas but found at similar levels to nondiabetic controls. This is in contrast to the established increased pancreatic enterovirus expression in T1D-associated autoantibody-positive individuals and in T1D, where viral infections contribute to abnormalities in both the endocrine and exocrine pancreas (51).

Enteroviral RNA was detected and quantified by the highly sensitive smFISH method, which had originally been developed to visualize each mRNA molecule as a computationally identifiable fluorescent spot by fluorescence microscopy (62). We had adapted smFISH for enteroviral RNA screening in the pancreas and called all positive spots “puncta” (51, 53). By using this method, Farack et al. identified transcriptional heterogeneity of β cells in the pancreas with some β cells containing much more insulin, which they called “extreme” β cells. In these cells, insulin mRNA could not be distinguished as puncta anymore but as large-signal accumulation (63). Similarly, viral RNA is seen as small fluorescent spots within cells, which were separately counted for each cell by moving the z-focus of the microscope through each virus^+^ cell. Cells were defined as “single infected” with 1–10 puncta or “fully infected” with ≥10 puncta. In the latter, viral RNA had highly accumulated in the cell and could no longer exactly be counted as single puncta. Therefore, we had developed such threshold analysis of low and highly infected cells.

Comparison of our results with earlier studies which had analysed coxsackieviruses in the T2D diabetes pancreas reveals several important differences (1): the presence of virus in these studies was limited to viral capsid VP1 staining, which has a much lower sensitivity (53) and specificity (64) (2); VP1 had exclusively been analysed within islets, where viruses are very rare and the number and size of islets very heterogeneous; and (3) VP1 positivity in islets of each donor had only been based on qualitative results, and not on viral quantification, which excluded stringent statistical analysis.

While viral^+^ cells within islets are a rare phenomenon, they were seen more frequently in proximity to the islets, that is, within three layers next to insulin^+^ β cells in T1D and, even in this analysis in T2D, viral RNA^+^ cells were 35-fold more frequent in the islet periphery than within islets and fivefold more than in the exocrine pancreas (then normalized to the respective area as presented in **Figures 1D–F**). Previous analyses suggest that such “peripheral cells” (either within or near islet cells) are more associated with a pathological phenotype than other islet cells. For example, using large-scale electron microscopy images (“nanotomy”) of nPOD human pancreas tissue, de Boer et al. identified morphologically abnormal cells containing both endocrine and exocrine granules in organ donors with T1D. These cells could neither been characterized as endocrine nor as exocrine cells (65). Also, two important studies show β-cell heterogeneity markers with their expression reduced frequently at the islet periphery. Van der Meulen et al. observed unusual immature “virgin” urocortin (UCN)3-negative β cells at the islet periphery. While labeling specific plastic cells, which undergo transdifferentiation, UCN3 is one of the first β-cell genes, which is downregulated during β-cell failure and, thus, also marks dysfunctional and dedifferentiated β-cells (66, 67). Another heterogeneity gene, ST8Si1, is often seen lost at the islet periphery, although such ST8Si1^−^ β cells are highly functional (68), and ST8Si1 expression is increased in T2D (68), possibly as part of the sialic acid-mediated immune response (69).

As viral infections promote β-cell dysfunction and dedifferentiation (70), several scenarios of the mechanisms of viral RNA presence in a subpopulation of cells in close islet neighborhood are possible; either infected cells have lost endocrine hormone expression and dedifferentiated, their specific phenotype makes them more vulnerable to viral infection or they hide from the immune system and thus remain a long time in the system. This may be a major path to diabetes pathology and remains to be investigated.

Independent of their diabetes state, it became apparent that most donors with a higher number of virus^+^ cells in the pancreas (>20) were from the age group 45+ years. With the age-dependent reduction in the immune response (71), it is possible that enteroviruses are not fully cleared in older individuals. Such hypothesis is in line with the increased vulnerability to infectious as well as metabolic diseases at an older age (71), and with a chronic low-grade inflammation, together referred to as “inflamm-aging.”

An overall existence of a low grade persistent viral infection in the pancreas may contribute to the progression of β-cell destruction and T2D in vulnerable individuals over time. Enteroviral RNAs trigger long-term pathology in the heart such as cardiac dysfunction and cardiomyopathy (72), both part of the metabolic syndrome and T2D. While acute viral infection requires viral clearance through the immune system, viral RNAs remain persistently in few cells and may cause pathology in genetically predisposed individuals. If not primarily, it could trigger potentiation of inflammation. For example, MafA, a crucial transcription factor for β-cell function is remarkably decreased in T2D β cells and its reduction leads to critical changes in the β-cell anti-viral response and susceptibility to enterovirus infection (73). In response, levels of MafA and other β-cell functional markers are further reduced by β-cell dysfunction and inflammatory stress, which then leads to a vicious cycle with diabetes progression eventually. These mechanistical pathways came from *in-vitro* studies, in which virus effects could be studied directly.

Although we do not see differences in enteroviral RNA disposition in the pancreas from nondiabetic donors and those with T2D, the inflammatory process induced by infections during life may contribute to β-cell failure through various mechanisms and progression to T2D at an older age.

## Author’s note

The content and views expressed are the responsibility of the authors and do not necessarily reflect the official view of nPOD. Organ Procurement Organizations (OPO) partnering with nPOD to provide research resources are listed at https://npod.org/for-partners/npod-partners/.”

## Data availability statement

The original contributions presented in the study are included in the article/**Supplementary Material**. Further inquiries can be directed to the corresponding author.

## Ethics statement

Ethical approval for the use of human pancreatic tissue had been granted by the Ethics Committee of the University of Bremen. The study complied with all relevant ethical regulations for work with human tissue for research purposes. Organ donors are not identifiable and anonymous, such approved analyses using tissue for research is covered by the NIH Exemption 4 (Regulation PHS 398). Organ donors or next of kin provided written informed consent for organ donation for research (46).

## Author contributions

HL performed experiments, analysed data and wrote the paper. SG designed and performed experiments and analysed data. AG, SS performed experiments, analysed data. AP provided intellectual support, pathological specimen and demographic data. KM designed experiments, analysed data, supervised the project and wrote the paper.

## Supplementary material

Supplementary Figure 1 Comparison of results from this study with pancreases from organ donors with T1D. Detection and quantification of viral RNA in FFPE pancreases from control donors without diabetes (*n* = 15), and donors with T2D (*n* = 29) from of this study and comparison with a previous study (51) of FFPE pancreases from donors with T1D (*n* = 15). The dashed box shows the T2D donor with >900 enteroviral RNA^+^ cells. **P* < 0.05 by Mann–Whitney non-parametric two-tailed test.

Supplementary Figure 2 No correlation of enteroviral^+^ cells with β-cell area and age. In each graph, the number of all enteroviral RNA^+^ cells throughout the whole pancreas section (see ) were correlated with **(A)** the number of islets, **(B)** β-cell area, and **(C)** donor age. **(A–C)** All control (*n* = 15) and T2D (*n* = 29) organ donors of this study were included together with previously analyzed control nondiabetic organ donors from a younger cohort **(C)** *n* = 14; mean age of 21 years). The dashed boxes show donors with >20 enteroviral RNA^+^ cells. Each individual point of the scatter graphs represents the mean of two technical replica from each donor pancreas, boxes are means ± SEM from all donors.

Supplementary Figure 3 Subgroup analyses of donors with T2D and single Aab^+^ (from data in ). The T2D cohort was divided into Aab^−^ (*n* = 17) and single Aab^+^ (*n* = 12) cases and subgroup analyses performed; each of the two subgroups was compared to the nondiabetic control pancreases (*n* = 15). **(A)** Detection and quantification of viral RNA in FFPE pancreases presented as **(A)** mean number of all enteroviral RNA^+^ cells throughout the whole pancreas section. **(B)** All enteroviral mRNA^+^ cells were normalized to the whole pancreas area of the respective section. **(C)** Enteroviral mRNA^+^ cells within islets were normalized to islet area (insulin^+^ stained area in mm^2^). **(D)** Quantification of enteroviral RNA^+^/CD45 co-positive cells throughout the whole pancreas section and **(E)** of CD45^+^ cells within insulin containing islets normalized to mm^2^ islet area. **(F)** For β-cell area analysis, the percentage of β cells were calculated by the ratio of mm^2^ insulin area and mm^2^ of the whole pancreas area from each section (previously also called β-cell volume). Each individual point of the scatter graphs represents the mean of two technical replica from each donor pancreas, boxes are means ± SEM from all donors. **P* < 0.05 by Mann–Whitney non-parametric two-tailed test, in which either the Aab^–^T2D group or the Aab^+^-T2D group was compared to the control group without diabetes.
