# Supplementary figures and images for: Enteroviral infections are not associated with type 2 diabetes
Source: Front Endocrinol (Lausanne). 2023 Oct 30;14:1236574. doi: 10.3389/fendo.2023.1236574 (PMC10643152; doi:10.3389/fendo.2023.1236574)

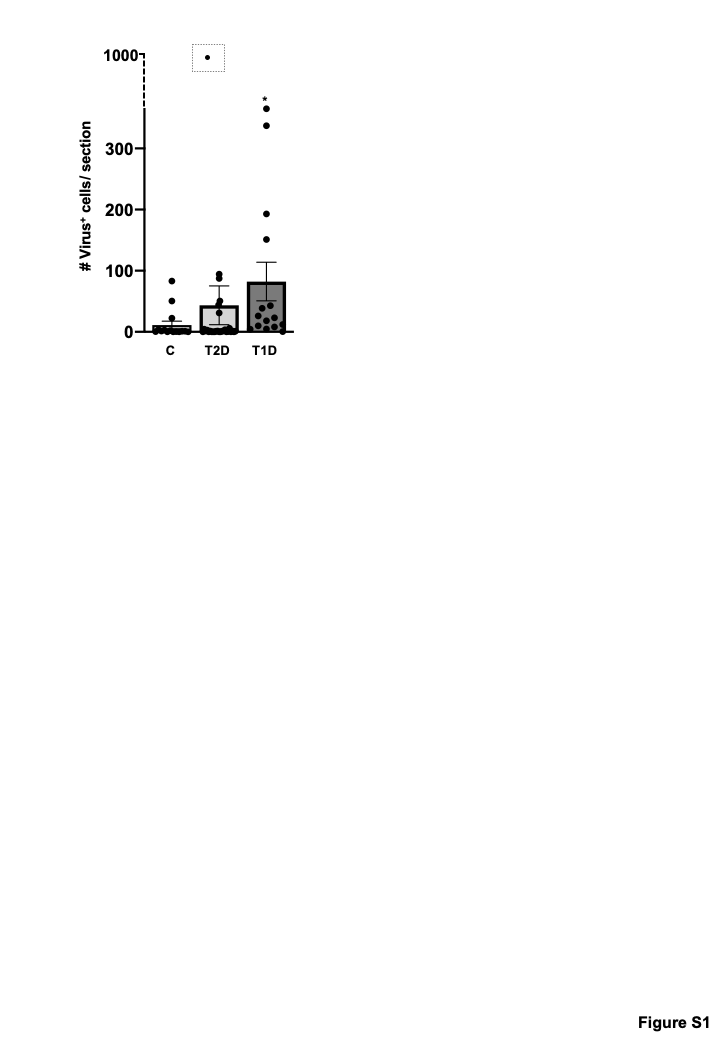

Supplement: Supplementary Figure 1 — Comparison of results from this study with pancreases from organ donors with T1D. Detection and quantification of viral RNA in FFPE pancreases from control donors without diabetes (n = 15), and donors with T2D (n = 29) from of this study and comparison with a previous study (51) of FFPE pancreases from donors with T1D (n = 15). The dashed box shows the T2D donor with >900 enteroviral RNA+ cells. *P < 0.05 by Mann–Whitney non-parametric two-tailed test. [file Image_1.tiff]

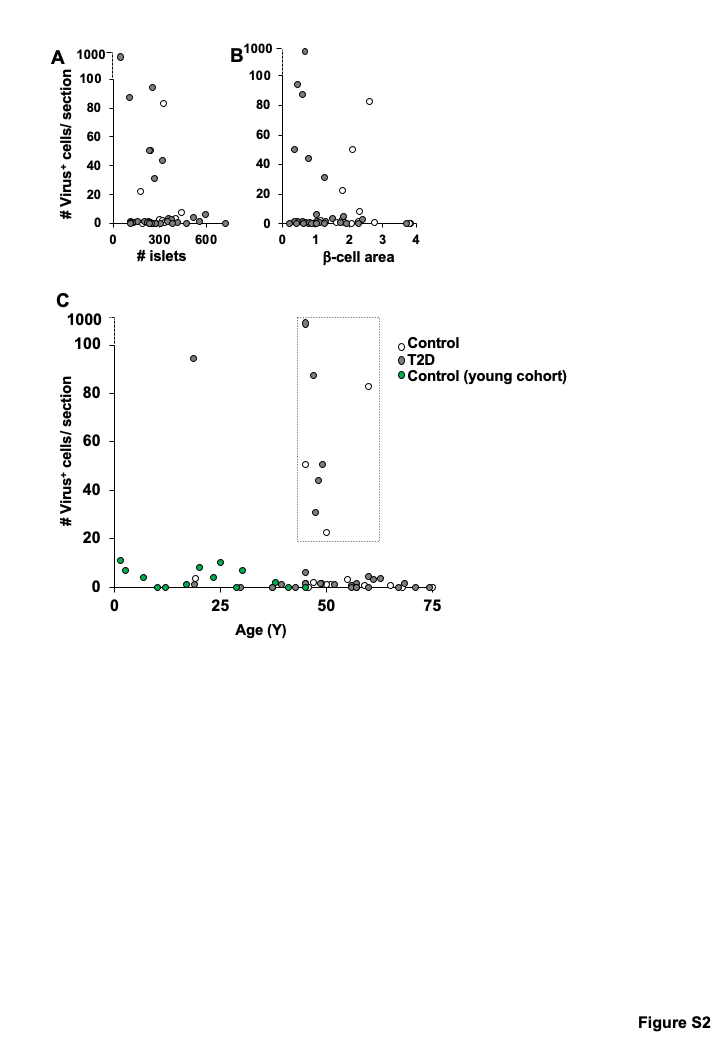

Supplement: Supplementary Figure 2 — No correlation of enteroviral+ cells with β-cell area and age. In each graph, the number of all enteroviral RNA+ cells throughout the whole pancreas section (see ) were correlated with (A) the number of islets, (B) β-cell area, and (C) donor age. (A–C) All control (n = 15) and T2D (n = 29) organ donors of this study were included together with previously analyzed control nondiabetic organ donors from a younger cohort (C) n = 14; mean age of 21 years). The dashed boxes show donors with >20 enteroviral RNA+ cells. Each individual point of the scatter graphs represents the mean of two technical replica from each donor pancreas, boxes are means ± SEM from all donors. [file Image_2.tiff]

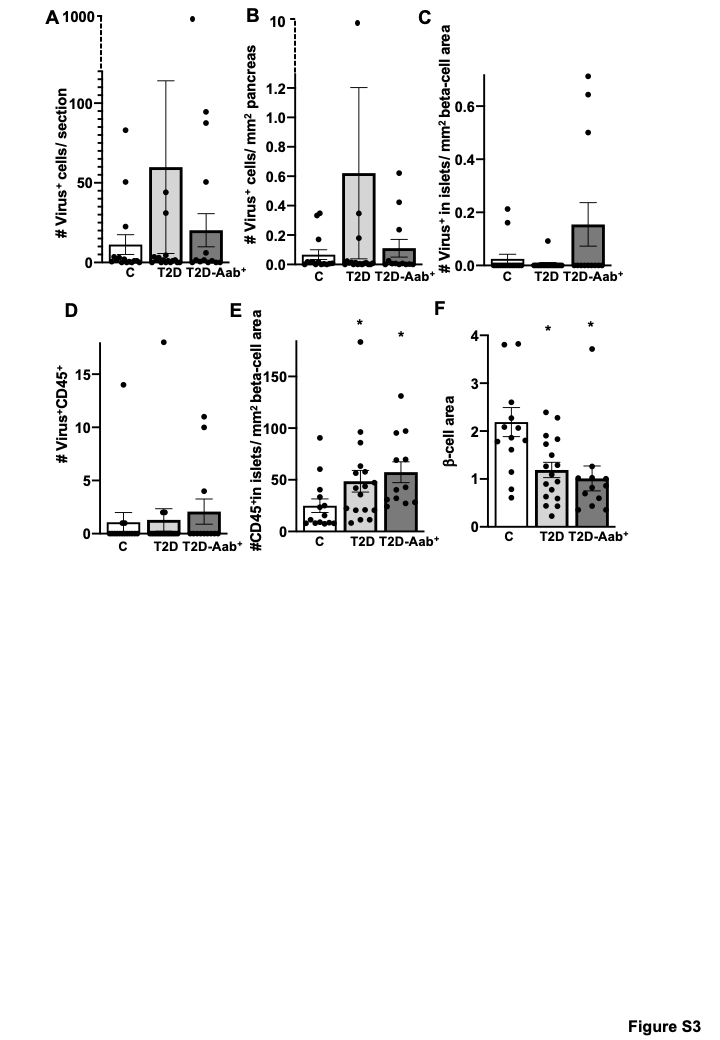

Supplement: Supplementary Figure 3 — Subgroup analyses of donors with T2D and single Aab+ (from data in ). The T2D cohort was divided into Aab− (n = 17) and single Aab+ (n = 12) cases and subgroup analyses performed; each of the two subgroups was compared to the nondiabetic control pancreases (n = 15). (A) Detection and quantification of viral RNA in FFPE pancreases presented as (A) mean number of all enteroviral RNA+ cells throughout the whole pancreas section. (B) All enteroviral mRNA+ cells were normalized to the whole pancreas area of the respective section. (C) Enteroviral mRNA+ cells within islets were normalized to islet area (insulin+ stained area in mm2). (D) Quantification of enteroviral RNA+/CD45 co-positive cells throughout the whole pancreas section and (E) of CD45+ cells within insulin containing islets normalized to mm2 islet area. (F) For β-cell area analysis, the percentage of β cells were calculated by the ratio of mm2 insulin area and mm2 of the whole pancreas area from each section (previously also called β-cell volume). Each individual point of the scatter graphs represents the mean of two technical replica from each donor pancreas, boxes are means ± SEM from all donors. *P < 0.05 by Mann–Whitney non-parametric two-tailed test, in which either the Aab–T2D group or the Aab+-T2D group was compared to the control group without diabetes. [file Image_3.tiff]
