# Supplementary material for: Enteroviral infections are not associated with type 2 diabetes
Source: Front Endocrinol (Lausanne). 2023 Oct 30;14:1236574. doi: 10.3389/fendo.2023.1236574 (PMC10643152; doi:10.3389/fendo.2023.1236574)
Supplement: Supplementary file 4 [file Table_1.pdf]

Suppl.Table 1. nPOD inventory donor case IDs and demographics.

	ID	type	Aab	Gender	Ethnicity	DurDiabetes	HbA1c	C-pep.	CoD
1	6020	control	Aab-	male	Caucasian	0		2.82	Cerebrovascular/Stroke
2	6102	control	Aab-	female	Caucasian	0	6.1	0.55	Cerebrovascular/Stroke
3	6008	control	Aab-	female	Caucasian	0			head Trauma
4	6279	control	Aab-	male	Caucasian	0		8.01	Head Trauma
5	6288	control	Aab-	male	Caucasian	0		12.96	Head Trauma
6	6295	control	Aab-	female	African Am	0	5.3	10.91	Cerebrovascular/Stroke
7	6009	control	Aab-	male	Caucasian	0		11.32	Anoxia
8	6168	control	Aab-	male	Hispanic	0	6.2		Cerebrovascular/Stroke
9	8003	control	Aab-	male		0			Cerebrovascular/Stroke
10	6013	control	Aab-	male	Caucasian	0		2.80	Cerebrovascular/Stroke
11	6017	control	Aab-	female	Caucasian	0		9.89	Cerebrovascular/Stroke
12	6165	control	Aab-	female	Caucasian	0	5.6	4.45	Cerebrovascular/Stroke
13	6022	control	Aab-	male	Caucasian	0		4.99	Cerebrovascular/Stroke
14	6012	control	Aab-	female	Caucasian	0		2.97	Cerebrovascular/Stroke
15	6545	control	Aab-	female	Caucasian	0		1.78	Cerebrovascular/Stroke
**MEAN**				**7F/8M**		**0.0**	**5.8**	**6.1**	
1	6133	T2D	Aab-	female	Caucasian	20		0.84	Anoxia
2	6277	T2D	Aab-	male	African Am	10		0.47	Cerebrovascular/Stroke
3	6194	T2D	Aab-	male	Caucasian	13	7.3	0.16	Cerebrovascular/Stroke
4	6206	T2D	Aab-	male	Caucasian	10	8.5	11.15	Cerebrovascular/Stroke
5	6191	T2D	Aab-	female	Caucasian	10	6.0	6.14	Cerebrovascular/Stroke
6	6221	T2D	Aab-	female	Caucasian	4		3.05	Cerebrovascular/Stroke
7	6273	T2D	Aab-	female	African American	2		3.17	Anoxia
8	6272	T2D	Aab-	female	African American	10		7.55	Anoxia
9	6186	T2D	Aab-	male	Caucasian	5	6.3	2.98	Cerebrovascular/Stroke
10	6059	T2D	Aab-	female	Hispanic	0.25		10.68	Anoxia
11	6304	T2D	Aab-	female	Hispanic	25		2.34	Cerebrovascular/Stroke
12	6157	T2D	Aab-	female	African Am	1	6.9	2.74	Cerebrovascular/Stroke
13	6114	T2D	Aab-	male	Caucasian	2	7.8	0.58	Anoxia
14	6139	T2D	Aab-	female	Hispanic	1.5		0.6	Anoxia
15	6132	T2D	Aab-	female	Hispanic	0	9.1	0.80	Anoxia
16	6259	T2D	Aab-	male	Caucasian	10		1.31	Cerebrovascular/Stroke
17	6541	T2D	Aab-	male	Hispanic	1	11.1	3.41	Cerebrovascular/Stroke
18	6499	T2D	mIAA+*	male	Caucasian	3	10.7	0.96	Head Trauma
19	6280	T2D	mIAA+*	male	African Am	10		3.71	Cerebrovascular/Stroke
20	6329	T2D	mIAA+*	female	Hispanic	25	11.2	7.46	Anoxia
21	6249	T2D	mIAA+*	female	Asian	15		4.17	Cerebrovascular/Stroke
22	6109	T2D	mIAA+	female	Hispanic	0	8.0	0.025	Cerebrovascular/Stroke
23	6189	T2D	mIAA+*	female	Caucasian	26		1.85	Cerebrovascular/Stroke
24	6149	T2D	GADA+	female	African American	16		11.55	Cerebrovascular/Stroke
25	6283	T2D	mIAA+*	female	Caucasian	17	9.6	1.77	Cerebrovascular/Stroke
26	6300	T2D	GADA+	male	Hispanic	0	6.5	3.19	Anoxia
27	6297	T2D	mIAA+*	male	Caucasian	3		3.23	Anoxia
28	6142	T2D	mIAA+*	female	Hispanic	14		0.19	Infectious Disease - Bacterial Meningitis
29	6269	T2D	mIAA+*	male	African Am	5		13.91	Cerebrovascular/Stroke
MEAN				**17F/12M**		**8.9**	**8.4**	**3.8**	

Suppl.Table 2. Number of viral mRNA positive cells in the pancreas. Related to Fig.1. Mean number of all viral mRNA positive cells/slide in each donor throughout the pancreas and separated in cells with the appearance of ≥10 (full grade infection) or 1-9 single puncta per cell (low grade infection)

	ID	type	Virus (n)	full grade(≥10)	low grade (<10)
1	6020	control	83	78	5
2	6102	control	50.5	45	5.5
3	6008	control	22.5	22	0.5
4	6279	control	3.5	3.5	0
5	6288	control	3	3	0
6	6295	control	2	2	0
7	6009	control	1.5	1.5	0
8	6168	control	1	1	0
9	8003	control	1	1	0
10	6013	control	0.5	0.5	0
11	6017	control	0.5	0.5	0
12	6165	control	0	0	0
13	6022	control	0	0	0
14	6012	control	0	0	0
15	6545	control	0	0	0
**MEAN**			**11.3**	**10.5**	**0.7**
1	6133	T2D	925	893	32
2	6277	T2D	44	43	1
3	6194	T2D	31	30.5	0.5
4	6206	T2D	4.5	4.5	0
5	6191	T2D	3.5	3.5	0
6	6221	T2D	3	3	0
7	6273	T2D	1.5	1.5	0
8	6272	T2D	1.5	1.5	0
9	6186	T2D	1.5	1.5	0
10	6059	T2D	1	1	0
11	6304	T2D	1	1	0
12	6157-03	T2D	0	0	0
13	6114-08	T2D	0	0	0
14	6139	T2D	0	0	0
15	6132	T2D	0	0	0
16	6259	T2D	0	0	0
17	6541	T2D	0	0	0
18	6499	T2D	94.5	88	6.5
19	6280	T2D	87.5	83	4.5
20	6329	T2D	50.5	44	6.5
21	6249	T2D	6	5.5	0.5
22	6109	T2D	1.5	1.5	0
23	6189	T2D	1.5	1.5	0
24	6149	T2D	1	1	0
25	6283	T2D	0.5	0.5	0
26	6300	T2D	0	0	0
27	6297	T2D	0	0	0
28	6142	T2D	0	0	0
29	6269	T2D	0	0	0
MEAN			**43.5**	**41.7**	**1.8**
